# Endometriotic Peritoneal Fluid Promotes Myofibroblast Differentiation of Endometrial Mesenchymal Stem Cells

**DOI:** 10.1155/2019/6183796

**Published:** 2019-06-02

**Authors:** Zhenzhen Zhang, Luxuan Suo, Yabing Chen, Li Zhu, Guiping Wan, Xiaodong Han

**Affiliations:** ^1^Immunology and Reproduction Biology Laboratory & State Key Laboratory of Analytical Chemistry for Life Science, Medical School, Nanjing University, Nanjing 210093, China; ^2^Jiangsu Key Laboratory of Molecular Medicine, Nanjing University, Nanjing 210093, China; ^3^Affiliated Hospital of Integrated Traditional Chinese and Western Medicine, Nanjing University of Chinese Medicine, Nanjing 210028, China; ^4^Jiangsu Province Academy of Traditional Chinese Medicine, Nanjing 210028, China

## Abstract

During the development of endometriosis, the presence of fibrotic tissues in and surrounding endometriotic lesions may lead to subsequent adhesion, anatomic distortion, and chronic pain. Therefore, studies aimed at clarifying the underlying mechanisms of fibrogenesis in endometriosis could potentially provide a novel strategy for effective treatment. Mesenchymal stem cells (MSCs) play a key role in fibrotic diseases by differentiating into myofibroblasts in appropriate microenvironment. In this study, we collected endometrial and endometriotic tissues from patients with endometriosis (*n* = 32) and control patients without endometriosis (*n* = 20) to compare the expression of fibrotic proteins and investigate the effect of endometriotic peritoneal fluid (PF) on myofibroblast differentiation of endometrial MSCs. We found that the expression of fibrotic proteins, including alpha-smooth muscle actin (*α*-SMA), type I collagen (collagen I), connective tissue growth factor (CTGF), and fibronectin, and the extent of fibrosis extremely enhanced in ectopic endometria compared with eutopic endometria from the same patients with endometriosis and normal endometria from patients without endometriosis. We next isolated and identified endometrial MSCs and found that treatment with endometriotic PF strongly induced endometrial MSCs to differentiate into myofibroblasts concomitant with the activation of Smad2/3. Moreover, ectopic endometrial MSCs expressed elevated collagen I, *α*-SMA, fibronectin, and CTGF. Sushi domain containing-2 (SUSD2), a marker of endometrial MSCs, and *α*-SMA, a well-recognized marker for myofibroblasts, colocalized extensively in ectopic endometria while seldom in normal and eutopic endometria. These findings suggest that ectopic endometrial MSCs are probably more susceptible to myofibroblast differentiation because of the long-term influence of endometriotic PF. All together, we report for the first time that endometriotic PF promotes myofibroblast differentiation of endometrial MSCs. This understanding will greatly improve our understanding of the pathophysiology of endometriosis and help design better therapeutics.

## 1. Introduction

Endometriosis, characterized by the presence of endometrial tissue outside the uterus, is a common gynecologic disease that affects 10% to 15% of all women and 35% to 50% of women with pelvic pain and/or infertility [1, 2]. During the development of endometriosis, the presence of fibrotic tissues in and surrounding the endometriotic lesions may lead to subsequent adhesion, anatomic distortion, and chronic pain [3, 4]. Therefore, studies aimed at clarifying the underlying mechanisms of fibrogenesis in endometriosis could potentially provide a novel strategy for effective treatment. Several studies have reported the presence of fibromuscular tissue and fibrotic proteins in endometriosis from a histological viewpoint [5–8]. On the other hand, some groups have attempted to explore the mechanisms of fibrogenesis in endometriosis based on cytological experiments in vitro [9–11]. However, inconsistent fibrotic markers are often examined by different groups in aforementioned two types of studies, limiting integration of the results and further research.

Although the pathogenesis of endometriosis remains elusive, growing evidences have demonstrated that endometrium-derived mesenchymal stem cells (MSCs) and epithelial progenitors are crucial in the pathogenesis of endometriosis [12–14]. It is considered as the main cause for endometriotic implants that endometrial MSCs and epithelial progenitors shed through the fallopian tube into the peritoneal cavity during menses [15]. It has been reported that ectopic endometrial MSCs have a higher proliferation potential in vitro and form more new blood vessels when transplanted into immunodeficient mice compared with matched eutopic samples [16, 17]. Suda et al. have suggested that clonal expansion of endometrial epithelial cells with cancer-associated mutations causes endometriosis.

Current evidences suggest that MSCs play a key role in fibrotic diseases [18]. It has been reported that bone marrow-, kidney-, and lung-derived MSCs are the precursors of myofibroblasts, which are proposed as the main source of extracellular matrix in fibrosis [19–21]. Since MSCs derived from various tissues have similar differentiative potential [22], it is reasonable to assume that endometrial MSCs may promote fibrogenesis in endometriosis by differentiating into myofibroblasts. However, to the best of our knowledge, little is known about myofibroblast differentiation of endometrial MSCs.

Moreover, the differentiative properties of MSCs are regulated by their surrounding microenvironment [23]. Ectopic endometrial MSCs exist in the peritoneal cavity. Various cytokines, growth factors, and angiogenic factors are abnormally expressed in endometriotic peritoneal fluid (PF) [24]. From this perspective, the different biological behaviors between ectopic and eutopic endometrial MSCs derived from the same patients [17] may result from the effects of endometriotic PF. Therefore, it is interesting to investigate whether endometriotic PF affect the differentiation of endometrial MSCs.

In the present study, we examined the extent of fibrosis and the expression of four fibrotic markers, consistent with the markers in subsequent cell experiments, in human endometriotic or endometrial tissues. We isolated and identified normal, eutopic and ectopic endometrial MSCs, respectively, and determined the effects of endometriotic PF on myofibroblast differentiation of endometrial MSCs. In addition, we compared the expression of *α*-SMA, collagen I, CTGF, and fibronectin among normal, eutopic, and ectopic endometrial MSCs and detected the colocalization of the markers of endometrial MSCs and myofibroblasts in human endometriotic or endometrial tissues.

## 2. Materials and Methods

### 2.1. Patients and Specimens

The study has been reviewed and approved by the Institutional Review Board of Jiangsu Province Hospital on Integration of Chinese and Western Medicine. Written informed consent was obtained from all human subjects. Thirty-two patients with laparoscopically and histopathologically confirmed ovarian endometriosis and twenty control women were included in this study. Control patients were surgically treated for benign gynecological conditions such as uterine leiomyoma and benign ovarian cyst, having no evidence of endometriosis at laparoscopy. All of the patients with leiomyoma in the present study had intramural and/or subserosal myomas. The patients with endometriosis were staged according to the revised American Fertility Society scoring system (American Society for Reproductive Medicine, 1996). All of the women included in this study had regular menstrual cycles and none of them had received hormonal treatment for at least 3 months prior to the surgery. The clinical characteristics of the patients were listed in Table 1. Samples of endometrial and endometriotic tissues (one endometriotic lesion from each patient with endometriosis) were collected for the study. PF was aspirated from the cul-de-sac (Douglas) immediately after the establishment of the pneumoperitoneum and before any laparoscopic manipulation. The tubes were not flushed before or after PF collection. Blood-contaminated PF was excluded.

### 2.2. Histology and Immunohistochemistry

Masson trichrome stain and immunohistochemistry were performed according to common protocols [20]. For immunohistochemistry, the primary antibodies were employed as follows: rabbit anti-collagen I, rabbit anti-*α*-SMA, mouse anti-fibronectin, and rabbit anti-CTGF (Abcam). The secondary antibodies incubated were horseradish peroxidase-conjugated goat anti-rabbit/mouse immunoglobulin G (Boster). All immunohistochemical photographs were analyzed using Image ProPlus (version 6.0, Media Cybernetics) as described previously [25].

### 2.3. Isolation and Identification of Endometrial MSCs

Firstly, normal, eutopic, and ectopic endometrial stromal cells were isolated as previously described [26]. The endometrial specimens were washed and minced into small pieces. Digestion of the tissues was performed at 37°C in the presence of 1 mg/mL collagenase I (Sigma) and 40 *μ*g/mL DNAse I (Sigma) in DMEM/F-12 (Gibco). Subsequently, supernatant was filtered through a 40 mm nylon mesh to remove undigested tissue pieces and epithelial cells. Afterwards, magnetic bead selection of endometrial MSCs was performed as reported elsewhere [27]. Freshly isolated endometrial MSCs were cultured with DMEM containing 10% MSC-qualified fetal bovine serum, 4% L-glutamine, and 1% penicillin and streptomycin (Gibco) and maintained in a humidified atmosphere of 95% air and 5% CO_2_ at 37°C. The cells were passaged when they reached 70%-90% confluence.

### 2.4. Flow Cytometric Analysis

The cultured endometrial MSCs were trypsinized and incubated with monoclonal antibodies at 4°C for 30 min. The antibodies used were fluorescein isothiocyanate- (FITC-) anti-CD34, FITC-anti-CD45, phycoerythrin- (PE-) anti-CD14, PE-anti-CD31, PE-anti-CD44, PE-anti-CD73, PE-anti-CD90, PE-anti-CD105, PE-anti-CD146, PE-anti-CD117, PE-anti-HLA-ABC, and PE-anti-HLA-DR (BD). FITC- or PE-isotype-matched immunoglobulin G was used as isotype control. After washed with PBS, the cells were analyzed by a flow cytometer (FACSCalibur, BD). At least 10,000 events were collected for further analysis.

### 2.5. Multipotent Differentiation

#### 2.5.1. Osteogenic Differentiation

The multipotent differentiation ability of endometrial MSCs for osteogenesis was examined according to the manufacturer's instructions of StemPro Osteogenesis Differentiation Kit (Gibco). Positive induction was detected by alkaline phosphatase and alizarin red stainings.

#### 2.5.2. Adipogenic Differentiation

The multipotent differentiation ability of endometrial MSCs for adipogenesis was examined according to the manufacturer's instructions of StemPro Adipogenesis Differentiation Kit (Gibco). Positive induction was detected by oil red staining of lipid vacuoles.

### 2.6. Clonogenicity Assay

The single-cell suspensions of cultured endometrial MSCs were seeded in triplicates at a clonal density of 50-100 cells/cm^2^ into six-well plates coated with fibronectin. The first media change was after the first week, and half media changes were done twice weekly thereafter. Colonies were monitored microscopically to ensure they were derived from single cells. After 2 weeks, cultures were fixed and stained with crystal violet. Clusters of ≥50 cells were counted, and the colony efficiency was determined.

### 2.7. PF

The PF was immediately cleared of cells and cell debris by centrifugation at 2000 rpm for 10 min at 4°C, filtered through a 0.22 *μ*m-pore size membrane and stored at -80°C. For this study, 10 PF from women with endometriosis (endometriotic PF, mean age 35 years, range 26-43) and 10 PF from women without endometriosis (control PF, mean age 34.8, range 24-44) in proliferative phase of menstrual cycle were thawed and pooled.

### 2.8. Western Blot Analysis

Immunofluorescence analysis was performed as described previously [26]. Specific antibodies against collagen I, *α*-SMA, fibronectin, CTGF, p-Smad3 (Abcam), Smad2/3, and p-Smad2 (Cell Signalling Technology) were used as primary antibodies. Secondary anti-rabbit and anti-mouse antibodies and Immobilon Western chemiluminescent HRP Substrate (Millipore) were used to visualize immunoactive bands.

### 2.9. Immunofluorescent Staining and Quantitative Colocalization Analysis

Immunofluorescence analysis was performed as described previously [20]. Rabbit anti-collagen I, mouse anti-*α*-SMA, mouse antifibronectin, rabbit anti-CTGF (Abcam), and rabbit anti-SUSD2 (Sigma-Aldrich) were employed as the primary antibodies. Alexa Fluor 488- or 594-conjugated goat anti-rabbit or anti-mouse antibody (Invitrogen) was used as a secondary antibody. The nuclei were stained with 4′, 6-diamidino-2-phenylindole (DAPI) (Sigma-Aldrich). The images were captured using a confocal fluorescence microscope (Olympus). The quantitative colocalization analysis was performed using Image ProPlus (version 6.0, Media Cybernetics), and Manders' Colocalization Coefficient (R) was acquired.

### 2.10. Cell Proliferation Assay

The effects of the PF on the proliferation of endometrial MSCs were evaluated by cell counting assays using Cell Counting Kit-8 (CCK-8, Vazyme). Cells were seeded at 6 × 10^3^ cells per well in a 96-well plate and cultured in serum-free medium containing indicated concentration of control PF or endometriotic PF for 24 h. The relative cell number was determined using CCK-8 according to the manufacturer's instructions. The OD (absorbance) value of each well was converted to relative cell number using a standard curve.

### 2.11. Statistical Analysis

In vitro data represent at least three independent experiments using cells from a minimum of three separate isolations. Data that followed a normal distribution were presented as means ± SD and analyzed by *t*-test or paired *t*-test, while data that were not normally distributed were presented as boxplots, in which the bottom and top of the box represent the lower and upper quartiles, respectively, the band near the middle of the box represents the median, and the ends of the whiskers represent the smallest and the largest nonoutlier observations, and subjected to Mann-Whitney *U*-test or Wilcoxon's matched pairs test. All these tests were performed using the statistical package SPSS 17.0. *P* < 0.05 was considered statistically significant.

## 3. Results

### 3.1. Ectopic Endometria Exhibit Enhanced Expression of Fibrotic Proteins and Fibrosis Degree

**Figure 1:**
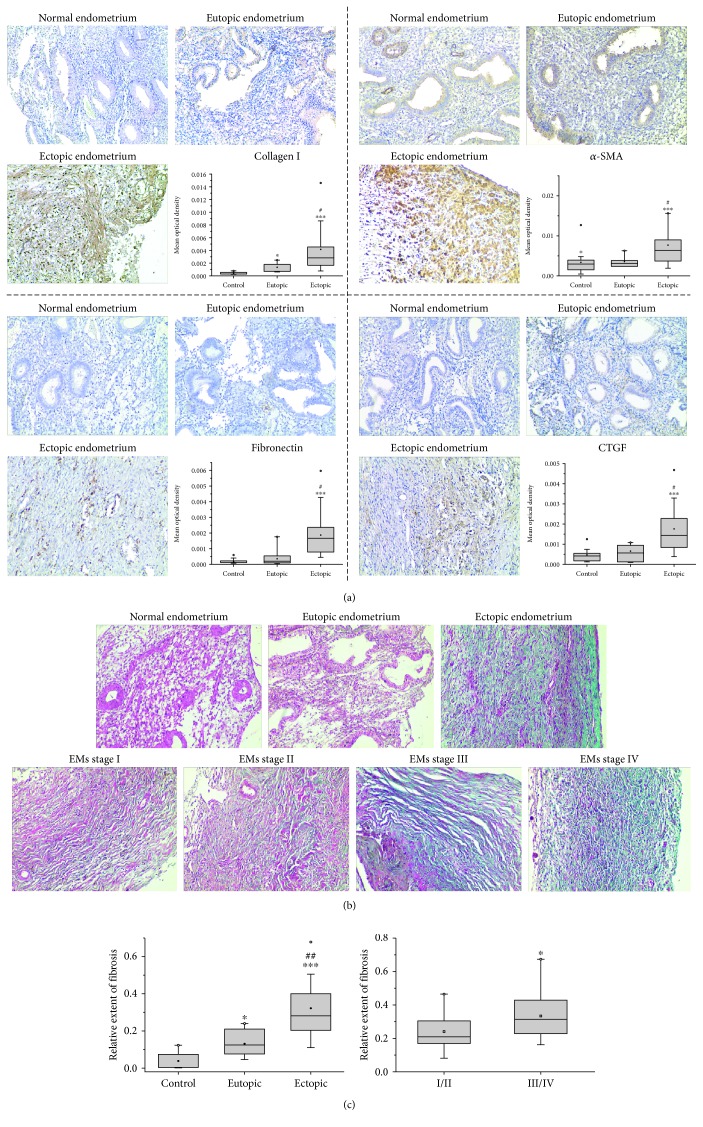
Ectopic endometria exhibit enhanced expression of fibrotic proteins and fibrosis degree. (a) The expression of collagen I, *α*-SMA, fibronectin, and CTGF in human endometriotic or endometrial tissues from 32 women with endometriosis and 20 women without endometriosis was detected by immunohistochemistry (×200) and quantified by Image ProPlus. ^∗^*P* < 0.05 versus the normal endometria. ^∗∗∗^*P* < 0.001 versus the normal endometria. ^#^*P* < 0.05 versus the paired eutopic endometria from the same patients with endometriosis. (b) The collagen deposits in the endometriotic or endometrial tissues were detected by Masson trichrome staining (×200). EMs represent endometriosis. (c) Relative extent of fibrosis was determined by quantifying the Masson trichrome staining by Image ProPlus. ^∗^*P* < 0.05 versus the normal endometria. ^∗∗∗^*P* < 0.001 versus the normal endometria. ^##^*P* < 0.01 versus the paired eutopic endometria from the same patients with endometriosis (A). ^∗^*P* < 0.05 versus the ectopic endometria from patients with stage I/II endometriosis (B).

As shown in Figure 1, the expression of collagen I, *α*-SMA, fibronectin, and CTGF and the extent of fibrosis in ectopic endometria were extremely enhanced compared with eutopic endometria from the same patients and normal endometria from patients without endometriosis. Moreover, the extent of fibrosis in endometriotic lesions from patients with stage III/IV endometriosis was markedly higher than that from patients with stage I/II endometriosis.

### 3.2. Isolation and Identification of Endometrial MSCs

**Figure 2:**
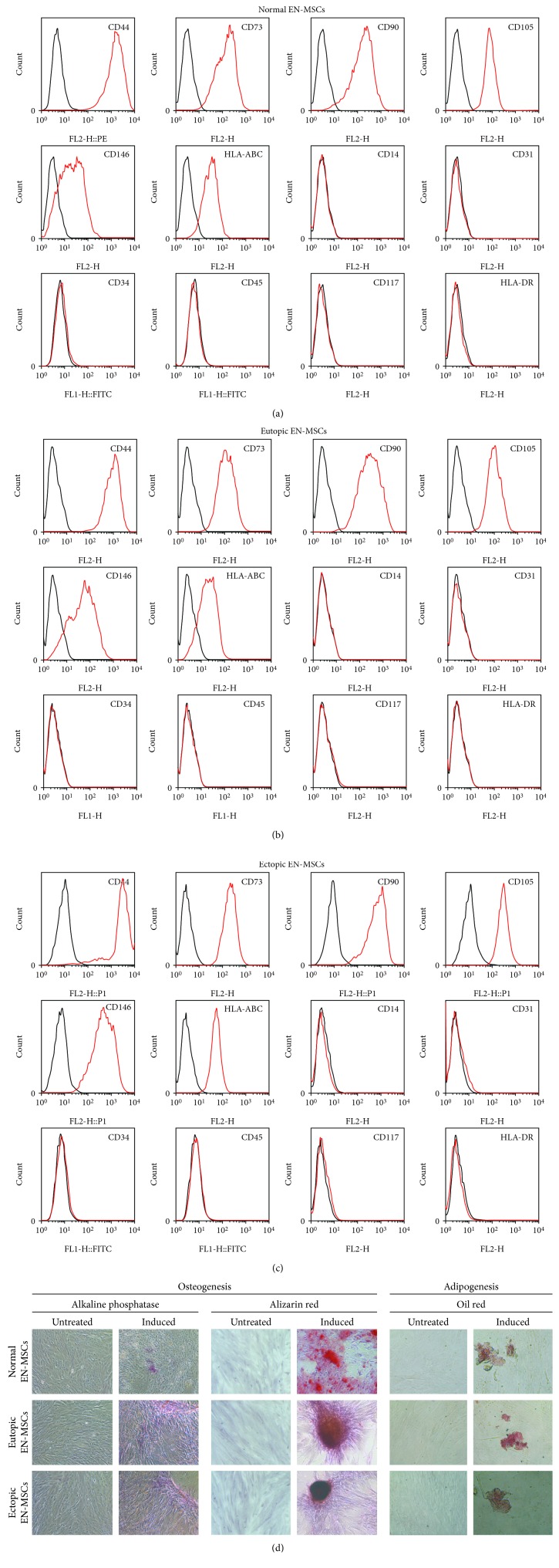
Isolation and identification of endometrial MSCs. (a-c) The normal, eutopic, and ectopic endometrial MSCs were identified for the expression of positive MSC markers CD44, CD73, CD90, CD105, CD146, and HLA-ABC and negative MSC markers CD14, CD31, CD34, CD45, CD117, and HLA-DR. Black lines, cells stained with a matched isotype control; red lines, cells stained with the antibodies indicated. EN-MSCs represent endometrial MSCs. (d) The normal, eutopic, and ectopic endometrial MSCs were identified for multipotency. The osteogenic differentiation was detected with alkaline phosphatase and alizarin red (×200), and the adipogenic differentiation was detected with Oil Red O (×400).

We isolated primary endometrial MSCs derived from normal, eutopic, and ectopic endometria, respectively, and investigated whether the subcultures of endometrial MSCs possessed fundamental properties of MSCs including immunophenotypic profile, multipotency, and clonogenicity. Flow cytometric analysis showed that the putative normal, eutopic, and ectopic endometrial MSCs expressed positive MSC markers CD44, CD73, CD90, CD105, CD146, and HLA-ABC and lacked negative MSC markers CD14, CD31, CD34, CD45, CD117, and HLA-DR (Figures 2(a)–2(c)). After treatment with respective differentiation-inducing medium, positive stainings for alkaline phosphatase and alizarin red were indicative of osteogenic differentiation. Evidence of adipogenic differentiation was found by the presence of intracellular lipid vacuoles when stained with Oil Red O (Figure 2(d)). The cloning efficiency was 0.83%-2.92%, which was similar to previous studies [28, 29]. Taken together, these isolated cells were confirmed as MSCs.

### 3.3. Endometriotic PF Promotes Myofibroblast Differentiation of Endometrial MSCs

**Figure 3:**
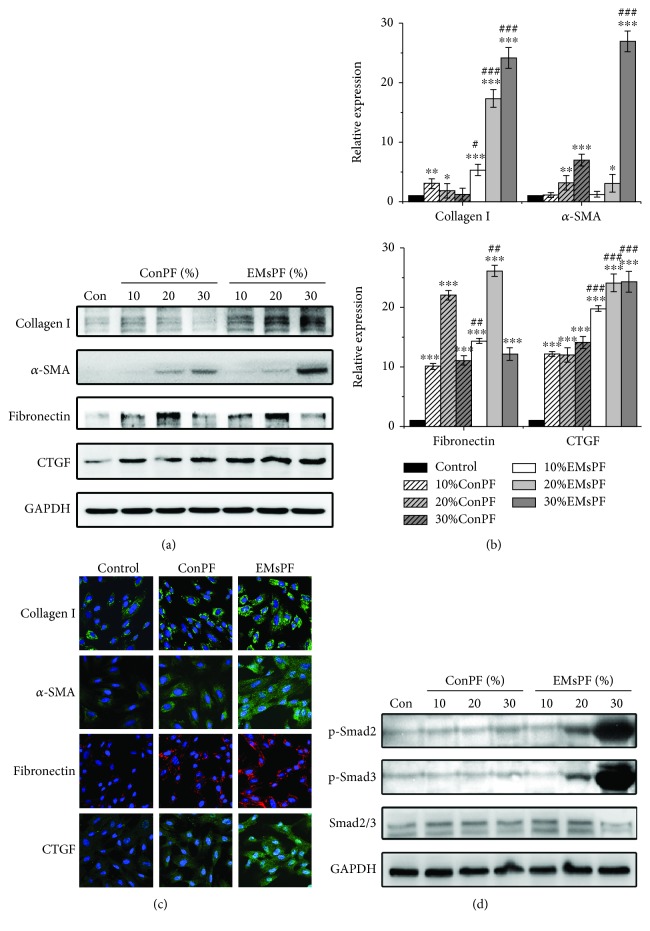
Endometriotic PF promotes myofibroblast differentiation of endometrial MSCs. (a) Endometrial MSCs from patients without endometriosis were treated with control PF or endometriotic PF, and the expression of collagen I, *α*-SMA, fibronectin, and CTGF was analyzed by Western blot. GAPDH was used as loading control. Con represents the cells treated without PF; ConPF and EMsPF represent the control PF and endometriotic PF, respectively. (b) The expression levels of proteins were quantified by densitometry and normalized to the expression of GAPDH. ^∗^*P* < 0.05 versus the cells treated without PF. ^∗∗^*P* < 0.01 versus the cells treated without PF. ^∗∗∗^*P* < 0.001 versus the cells treated without PF. ^#^*P* < 0.05 versus the cells treated with ConPF at the same concentration. ^##^*P* < 0.01 versus the cells treated with ConPF at the same concentration. ^###^*P* < 0.001 versus the cells treated with ConPF at the same concentration. (c) Endometrial MSCs from patients without endometriosis were treated with control or endometriotic PF, and the expression of collagen I, *α*-SMA, fibronectin, and CTGF was examined by immunofluorescence analysis. The nuclei were stained with DAPI (×600). (d) Endometrial MSCs from patients without endometriosis were treated with control or endometriotic PF, and the expression of p-Smad2, p-Smad3, and Smad2/3 was analyzed by Western blot. GAPDH was used as loading control.

Since the differentiative properties of MSCs are regulated by the microenvironment and endometriotic lesions were surrounded by PF [23, 24], we speculated that myofibroblasts in ectopic lesions might differentiate from endometrial MSCs in the presence of endometriotic PF. We therefore determined the effects of control PF and endometriotic PF on the myofibroblast differentiation of normal endometrial MSCs derived from patients without endometriosis, respectively. As shown in Figures 3(a)–3(c), endometriotic PF enhanced the expression of collagen I, *α*-SMA, fibronectin, and CTGF in endometrial MSCs more significantly than control PF, indicating that endometriotic PF could strongly induce endometrial MSCs to differentiate into myofibroblasts. Since activation of Smad2 or Smad3 plays an important role in myofibroblast differentiation [30], we also examined whether endometriotic PF activated Smad2 or Smad3 in endometrial MSCs. The results demonstrated that endometriotic PF profoundly induced the activation of Smad2/3 in endometrial MSCs (Figure 3(d)).

### 3.4. Ectopic Endometrial MSCs Expressed Elevated Fibrotic Proteins Compared with Normal and Eutopic Endometrial MSCs

**Figure 4:**
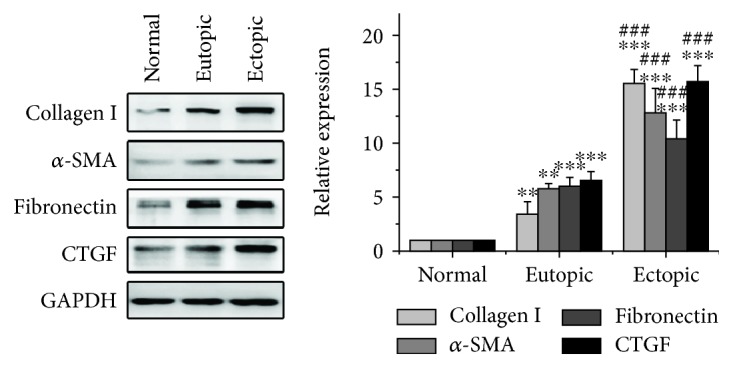
Ectopic endometrial MSCs expressed elevated fibrotic proteins compared with normal and eutopic endometrial MSCs. The expression of collagen I, *α*-SMA, fibronectin and CTGF was analyzed by Western blot. GAPDH was used as loading control. The expression levels of proteins were quantified by densitometry and normalized to the expression of GAPDH. ^∗∗^*P* < 0.01 versus normal endometrial MSCs. ^∗∗∗^*P* < 0.001 versus normal endometrial MSCs. ^###^*P* < 0.001 versus eutopic endometrial MSCs from the same patients with endometriosis.

In light of the findings in Figure 3, we speculated that ectopic endometrial MSCs probably expressed higher levels of fibrotic proteins than normal and eutopic endometrial MSCs because of long-term exposure to endometriotic PF. We therefore compared the expression of fibrotic proteins in the cells mentioned above and found that the expression of collagen I, *α*-SMA, fibronectin, and CTGF in ectopic endometrial MSCs was robustly intensified compared with eutopic endometrial MSCs from the same patients and normal endometrial MSCs from women without endometriosis (Figure 4).

### 3.5. Colocalization of Endometrial MSCs and Myofibroblasts Extensively in Ectopic Endometria

**Figure 5:**
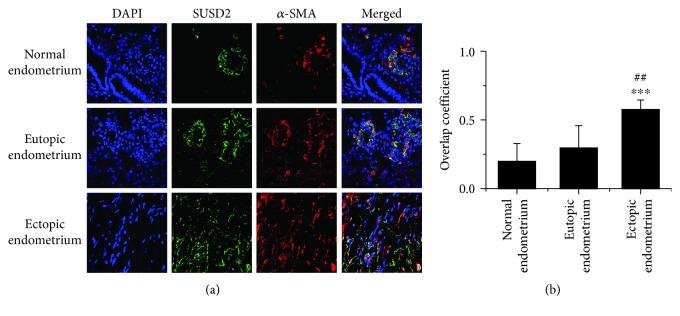
Colocalization of endometrial MSCs and myofibroblasts extensively in ectopic endometria. (a) The colocalization of SUSD2 and *α*-SMA in human endometriotic or endometrial tissues from women with or without endometriosis was examined by immunofluorescence analysis. The nuclei were stained with DAPI (×600). (b) The overlap coefficients were quantified by Image ProPlus. ^∗∗∗^*P* < 0.001 versus the normal endometria. ^##^*P* < 0.01 versus the paired eutopic endometria from the same patients with endometriosis.

We also examined the colocalization of SUSD2, a marker of endometrial MSCs, and *α*-SMA, a well-recognized marker for myofibroblasts in endometrial tissues. As shown in Figure 5, in normal endometria and eutopic endometria, immunofluorescent detection of SUSD2 and *α*-SMA showed their well-arranged perivascular localization. However, in ectopic endometria, both SUSD2 and *α*-SMA were dispersedly distributed. It is noteworthy that SUSD2 and *α*-SMA colocalized extensively in ectopic endometria while seldom in normal and eutopic endometria.

### 3.6. PF Exhibits a Minimal Effect on Proliferation of Endometrial MSCs

**Figure 6:**
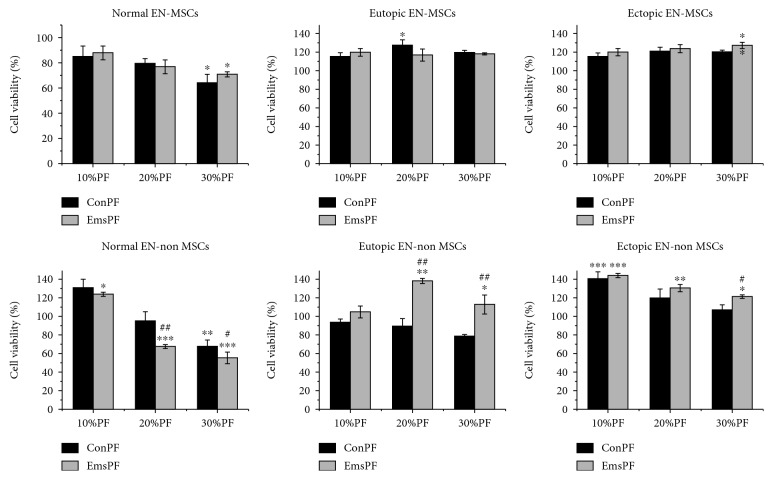
PF exhibits a minimal effect on the proliferation of endometrial MSCs. Endometrial MSCs or non-MSCs were treated with indicated concentrations of control or endometriotic PF for 24 h, and the cell viability was determined by CCK-8. EN-MSCs and EN-non MSCs represent endometrial MSCs and endometrial non-MSCs, respectively. ConPF and EMsPF represent control and endometriotic PF, respectively. ^∗^*P* < 0.05 versus the cells treated without PF. ^∗∗^*P* < 0.01 versus the cells treated without PF. ^∗∗∗^*P* < 0.001 versus the cells treated without PF. ^#^*P* < 0.05 versus the cells treated with ConPF at the same concentration. ^##^*P* < 0.01 versus the cells treated with ConPF at the same concentration.

Considering that endometrial MSCs, especially ectopic endometrial MSCs, have aberrant proliferative potential, we also evaluated the influence of PF on the proliferation of normal, eutopic, and ectopic endometrial MSCs, respectively. As shown in Figure 6, both control PF and endometriotic PF slightly decreased the viability of normal endometrial MSCs from patients without endometriosis while increased the viability of eutopic and ectopic endometrial MSCs from patients with endometriosis. However, there were no significant differences in the effects of control PF and endometriotic PF. In contrast, the viability of endometrial non-MSCs (SUSD2-) was dramatically affected by the PF. These results suggest that PF mainly modulated the differentiation but not proliferation of endometrial MSCs while affecting the proliferation of endometrial non-MSCs.

## 4. Discussion

One prominent histological feature of endometriosis is the presence of dense fibrotic tissue in and surrounding the lesions. *α*-SMA-positive myofibroblast-like cells or smooth muscle cells are frequently detected in the fibrotic areas associated with superficial peritoneal [31], ovarian [6, 7], and deep infiltrating endometriosis [5, 8]. Type I collagen has been reported as a major contributor to fibrosis in peritoneal endometriosis despite the presence of type I, III, and IV collagen in intrauterine and ectopic endometria of patients with endometriosis [32, 33]. In the present study, we compared the expression of fibrotic proteins as well as the extent of fibrosis in eutopic and ectopic endometria from patients with endometriosis and normal endometria from patients without endometriosis, which is absent in the previous studies. We found that the expression of *α*-SMA, collagen I, CTGF, and fibronectin and the extent of fibrosis in ectopic endometria were extremely higher than normal and eutopic endometria. Furthermore, our results showed that the extent of fibrosis in endometriotic lesions from patients with stage III/IV endometriosis was notably higher than stage I/II endometriosis, indicating that more advanced endometriosis was more fibrotic. Therefore, an important approach to understand the pathogenesis of endometriosis is to investigate the mechanisms of fibrogenesis in endometriotic lesions.

In recent years, MSCs isolated from various tissues such as bone marrow, kidney, and lung have been demonstrated to differentiate into myofibroblasts, suggesting that MSCs play a key role in the development of fibrotic diseases [19–21]. Meanwhile, the existence of MSCs with remarkable regenerative ability in endometrium has been proposed, and growing evidence shows that etiology of endometriosis involves endometrial MSCs [14]. In consideration of the similar differentiative potential of MSCs derived from various tissues and the crucial role of endometrial MSCs in the pathogenesis of endometriosis [22, 34], we assumed that endometrial MSCs may differentiate into myofibroblasts in the development of endometriosis.

We herein isolated and identified paired eutopic and ectopic endometrial MSCs from the same patients with endometriosis and normal endometrial MSCs from women without endometriosis in order to investigate the underlying mechanism for myofibroblast differentiation of endometrial MSCs in endometriosis. Several studies have indicated that PF from women with endometriosis has different expression patterns of numerous bioactive factors in comparison with PF from the control women [35, 36]. Therefore, we evaluated the influence of endometriotic PF and control PF on the expression of fibrotic proteins in endometrial MSCs, respectively. The endometrial MSCs treated with endometriotic PF had a greater ability to differentiate into a myofibroblast phenotype compared with the corresponding cells treated with control PF, as evidenced by the markedly strengthened expression of proteins involved in fibrogenesis. Moreover, the distinctively enhanced expression of fibrotic proteins in ectopic endometrial MSCs and the extensive colocalization of SUSD2 and *α*-SMA in endometriotic endometria suggest that ectopic endometrial MSCs are probably more susceptible to myofibroblast differentiation than normal and eutopic endometrial MSCs because of the long-term influence of endometriotic PF. Although different hypotheses, such as epigenetic regulation, could explain the altered functional properties detected in ectopic MSCs with respect to eutopic or normal MSCs [37], our data provide strong evidence that endometriotic PF, the microenvironment of endometriotic lesions, substantially give rise to myofibroblast differentiation of endometrial MSCs.

Nevertheless, it is undeniable that control PF could also promote the myofibroblast differentiation of endometrial MSCs in spite of the much lesser extent. A possible explanation is that there are several profibrogenic cytokines and growth factors, which are necessary for maintaining physiological function in control PF. On the other hand, our results also revealed that eutopic endometrial MSCs expressed more fibrotic proteins than normal endometrial MSCs, suggesting that the inherent characters of eutopic endometrial MSCs also contributed to the fibrogenesis in endometriosis. In addition, endometrial epithelial cells have been reported to promote fibrosis in endometriosis through epithelial-mesenchymal transition (EMT) [38]. PF from patients with endometriosis is rich in TGF-*β* and estrogen, both of which can promote EMT in endometriosis [39–43]. Therefore, it is speculated that endometriotic PF may play a role in the development of fibrosis in endometriosis by facilitating EMT.

In light of the notion that endometrial MSCs, once located in the microenvironment of the ectopic lesions, may undergo a selection process that could lead to the survival of the MSCs with enhanced proliferative property, we also assessed the influence of endometriotic PF on the proliferation of endometrial MSCs and found that endometriotic PF mainly modulated the differentiation of endometrial MSCs and affected the proliferation of endometrial non-MSCs. The results, together with the results in Figure 3, imply that endometriotic PF promotes endometrial MSCs to differentiate into myofibroblasts, and the differentiated endometrial non-MSCs acquire significantly increased proliferative capacity in the presence of endometriotic PF, consequently accelerating the growth of endometriotic lesions as well as fibrogenesis in it. Interestingly, the proliferation of eutopic endometrial MSCs was elevated by endometriotic PF much remarkably than ectopic endometrial MSCs derived from the same patients, while the proliferation of normal endometrial MSCs was inhibited in the same condition. These data suggest that there are some inherent differences between endometrial MSCs from patients with and without endometriosis facilitating the proliferation of eutopic but not normal endometrial MSCs in the presence of endometriotic PF, which is consistent with the theory of “eutopic endometrium determinism” [44].

## 5. Conclusions

In conclusion, we report for the first time that endometriotic PF promotes myofibroblast differentiation of endometrial MSCs, although the exact factors responsible for the profibrotic modulations still deserve further investigation. This understanding will greatly improve our understanding of the pathophysiology of endometriosis and help design better therapeutics.

## Figures and Tables

**Table 1:** Characteristics of recruited patients with endometriosis and controls.

	Endometriosis (*n* = 32)	Controls (*n* = 20)	*P* value
Age (year)			
Mean (SD)	34.4 (6.9)	35.0 (7.1)	0.756
Median (range)	34.5 (23-49)	33.5 (24-47)	
Menstrual phase			
Proliferative	16 (50)	11 (55)	0.726
Secretory	16 (50)	9 (45)	
Parity			
0	9 (28)	4 (20)	0.272
1	22 (69)	13 (65)	
≥2	1 (3)	3 (15)	
rASRM stage			
I	5 (16)	NA	NA
II	8 (25)		
III	9 (28)		
IV	10 (31)		

Data are *n* (%) unless stated otherwise. NA, not applicable; rASRM, revised American Society of Reproductive Medicine classification.

## Data Availability

The data used to support the findings of this study are included within the article.

## References

[1] Simoens S., Dunselman G., Dirksen C. (2012). The burden of endometriosis: costs and quality of life of women with endometriosis and treated in referral centres. *Human Reproduction*.

[2] Giudice L. C., Kao L. C. (2004). Endometriosis. *The Lancet*.

[3] Nisolle M., Donnez J. (1997). Peritoneal endometriosis, ovarian endometriosis, and adenomyotic nodules of the rectovaginal septum are three different entities. *Fertility and Sterility*.

[4] Matsuzaki S., Darcha C. (2014). Antifibrotic properties of epigallocatechin-3-gallate in endometriosis. *Human Reproduction*.

[5] van Kaam K. J. A. F., Schouten J. P., Nap A. W., Dunselman G. A. J., Groothuis P. G. (2008). Fibromuscular differentiation in deeply infiltrating endometriosis is a reaction of resident fibroblasts to the presence of ectopic endometrium. *Human Reproduction*.

[6] Liu X., Zhang Q., Guo S. W. (2017). Histological and immunohistochemical characterization of the similarity and difference between ovarian endometriomas and deep infiltrating endometriosis. *Reproductive Sciences*.

[7] Khare V. K., Martin D. C., Eltorky M. (1996). A comparative study of ovarian and pelvic wall-infiltrating endometriosis. *The Journal of the American Association of Gynecologic Laparoscopists*.

[8] Anaf V., Simon P., Fayt I., Noel J. (2000). Smooth muscles are frequent components of endometriotic lesions. *Human Reproduction*.

[9] Yuge A., Nasu K., Matsumoto H., Nishida M., Narahara H. (2007). Collagen gel contractility is enhanced in human endometriotic stromal cells: a possible mechanism underlying the pathogenesis of endometriosis-associated fibrosis. *Human Reproduction*.

[10] Matsuzaki S., Darcha C. (2013). Involvement of the Wnt/*β*-catenin signaling pathway in the cellular and molecular mechanisms of fibrosis in endometriosis. *PLoS One*.

[11] Zhang Q., Duan J., Liu X., Guo S. W. (2016). Platelets drive smooth muscle metaplasia and fibrogenesis in endometriosis through epithelial-mesenchymal transition and fibroblast-to-myofibroblast transdifferentiation. *Molecular and Cellular Endocrinology*.

[12] Dai L., Lou W., Zhu J., Zhou X., Di W. (2015). MiR-199a inhibits the angiogenic potential of endometrial stromal cells under hypoxia by targeting HIF-1*α*/VEGF pathway. *International Journal of Clinical and Experimental Pathology*.

[13] Gargett C. E., Schwab K. E., Zillwood R. M., Nguyen H. P. T., Wu D. (2009). Isolation and culture of epithelial progenitors and mesenchymal stem cells from human endometrium. *Biology of Reproduction*.

[14] Kao A. P., Wang K. H., Long C. Y. (2011). Interleukin-1*β* induces cyclooxygenase-2 expression and promotes the invasive ability of human mesenchymal stem cells derived from ovarian endometrioma. *Fertility and Sterility*.

[15] Gargett C. E., Masuda H. (2010). Adult stem cells in the endometrium. *Molecular Human Reproduction*.

[16] Moggio A., Pittatore G., Cassoni P., Marchino G. L., Revelli A., Bussolati B. (2012). Sorafenib inhibits growth, migration, and angiogenic potential of ectopic endometrial mesenchymal stem cells derived from patients with endometriosis. *Fertility and Sterility*.

[17] Kao A. P., Wang K. H., Chang C. C. (2011). Comparative study of human eutopic and ectopic endometrial mesenchymal stem cells and the development of an in vivo endometriotic invasion model. *Fertility and Sterility*.

[18] Kuppe C., Kramann R. (2016). Role of mesenchymal stem cells in kidney injury and fibrosis. *Current Opinion in Nephrology and Hypertension*.

[19] Kramann R., Schneider R. K., DiRocco D. P. (2015). Perivascular Gli1^+^ progenitors are key contributors to injury-induced organ fibrosis. *Cell Stem Cell*.

[20] Wang C., Gu S., Cao H. (2016). miR-877-3p targets Smad7 and is associated with myofibroblast differentiation and bleomycin-induced lung fibrosis. *Scientific Reports*.

[21] Sun Z., Wang C., Shi C. (2014). Activated Wnt signaling induces myofibroblast differentiation of mesenchymal stem cells, contributing to pulmonary fibrosis. *International Journal of Molecular Medicine*.

[22] Jiang Y., Jahagirdar B. N., Reinhardt R. L. (2002). Pluripotency of mesenchymal stem cells derived from adult marrow. *Nature*.

[23] Ma S., Xie N., Li W., Yuan B., Shi Y., Wang Y. (2014). Immunobiology of mesenchymal stem cells. *Cell Death & Differentiation*.

[24] Burney R. O., Giudice L. C. (2012). Pathogenesis and pathophysiology of endometriosis. *Fertility and Sterility*.

[25] Yin X., Xiang T., Li L. L. (2013). *DACT1*, an antagonist to Wnt/*β*-catenin signaling, suppresses tumor cell growth and is frequently silenced in breast cancer. *Breast Cancer Research*.

[26] Zhang Z., Cheng X., Gui T. (2016). Wenshen Xiaozheng Tang induces apoptosis and inhibits migration of ectopic endometriotic stromal cells. *Journal of Ethnopharmacology*.

[27] Ulrich D., Tan K. S., Deane J. (2014). Mesenchymal stem/stromal cells in post-menopausal endometrium. *Human Reproduction*.

[28] Rajaraman G., White J., Tan K. S. (2013). Optimization and scale-up culture of human endometrial multipotent mesenchymal stromal cells: potential for clinical application. *Tissue Engineering Part C: Methods*.

[29] Gurung S., Werkmeister J. A., Gargett C. E. (2015). Inhibition of transforming growth factor-*β* receptor signaling promotes culture expansion of undifferentiated human endometrial mesenchymal stem/stromal Cells. *Scientific Reports*.

[30] Zhou Y., Zhang Q., Gao Y. (2017). Induced pluripotent stem cell-conditioned medium suppresses pulmonary fibroblast-to-myofibroblast differentiation via the inhibition of TGF-*β*1/Smad pathway. *International Journal of Molecular Medicine*.

[31] Barcena de Arellano M. L., Gericke J., Reichelt U. (2011). Immunohistochemical characterization of endometriosis-associated smooth muscle cells in human peritoneal endometriotic lesions. *Human Reproduction*.

[32] Matsuzaki S., Canis M., Darcha C., Dechelotte P., Pouly J. L., Bruhat M. A. (1999). Fibrogenesis in peritoneal endometriosis. A semi-quantitative analysis of type-I collagen. *Gynecologic and Obstetric Investigation*.

[33] Stovall D. W., Anners J. A., Halme J. (1992). Immunohistochemical detection of type I, III, and IV collagen in endometriosis implants. *Fertility and Sterility*.

[34] Pittatore G., Moggio A., Benedetto C., Bussolati B., Revelli A. (2013). Endometrial adult/progenitor stem cells: pathogenetic theory and new antiangiogenic approach for endometriosis therapy. *Reproductive Sciences*.

[35] Wu M. H., Shoji Y., Wu M. C. (2005). Suppression of matrix metalloproteinase-9 by prostaglandin E_2_ in peritoneal macrophage is associated with severity of endometriosis. *The American Journal of Pathology*.

[36] Gazvani R., Templeton A. (2002). Peritoneal environment, cytokines and angiogenesis in the pathophysiology of endometriosis. *Reproduction*.

[37] Gerhardt K. (2016). Shedding light on endometriosis. *Biology of Reproduction*.

[38] Vigano P., Candiani M., Monno A., Giacomini E., Vercellini P., Somigliana E. (2018). Time to redefine endometriosis including its pro-fibrotic nature. *Human Reproduction*.

[39] Yang Y. M., Yang W. X. (2017). Epithelial-to-mesenchymal transition in the development of endometriosis. *Oncotarget*.

[40] Soni U. K., Chadchan S. B., Kumar V. (2018). A high level of TGF-B1 promotes endometriosis development via cell migration, adhesiveness, colonization, and invasiveness. *Biology of Reproduction*.

[41] Young V. J., Brown J. K., Saunders P. T. K., Duncan W. C., Horne A. W. (2014). The peritoneum is both a source and target of TGF-*β* in women with endometriosis. *PLoS One*.

[42] Du Y., Zhang Z., Xiong W. (2019). Estradiol promotes EMT in endometriosis via MALAT1/miR200s sponge function. *Reproduction*.

[43] Xu X., Othman E. E. D. R., Issaq H. J., Hornung D., al-Hendy A., Veenstra T. D. (2008). Multiplexed quantitation of endogenous estrogens and estrogen metabolites in human peritoneal fluid. *Electrophoresis*.

[44] Liang Y., Li Y., Liu K., Chen P., Wang D. (2015). Expression and significance of WNT4 in ectopic and eutopic endometrium of human endometriosis. *Reproductive Sciences*.

